# Short term and long term results after open *vs.* laparoscopic appendectomy in childhood and adolescence: a subgroup analysis

**DOI:** 10.1186/1471-2431-13-154

**Published:** 2013-10-01

**Authors:** Matthias Kapischke, Alexandra Pries, Amke Caliebe

**Affiliations:** 1 Department of Surgery, Klinik St. Georg, Lohmuehlenstrasse 05, D-20099 Hamburg, Germany; 2 Institute for Medical Informatics and Statistics, Bldg 31, University Hospital of Schleswig-Holstein, Campus Kiel, Arnold Heller Strasse 03, D-24105 Kiel, Germany

**Keywords:** Appendectomy, Paediatric surgery, Quality of life, SF-36

## Abstract

**Background:**

A comparative study was performed to compare quality of life after laparoscopic and open appendectomy in children and adolescents in a German General Hospital. The same study population was re-evaluated regarding their quality of life several years after operation.

**Methods:**

Children and adolescents (n = 158) who underwent appendectomy for acute appendicitis between 1999 and 2001 were retrospectively analysed. Seven years after surgery those patients were interviewed applying a SF-36 questionnaire regarding their quality of life.

**Results:**

For short term outcomes there was a trend towards reduced specific postoperative complications in the laparoscopically operated group (9.3 *vs.* 10.7%). Significantly more patients in the laparoscopic group would recommend the operation procedure to family members or friends than in the open group. Among the evaluated patients there was a significantly higher satisfaction concerning size and appearance of their scars in the laparoscopic group. The results of the evaluation in the eight categories of the SF-36 showed similar results in both groups.

**Conclusions:**

More patients with laparoscopic appendectomy appeared to be satisfied with their operation method as becomes evident by a higher recommendation rate and a higher satisfaction concerning their scars.

## Background

Laparoscopic appendectomy (LA) has been established during recent years as an option to open appendectomy (OA) in the treatment of acute appendicitis in children and adolescents. The clinical benefit is seen controversial; minor reduction of post operative complications and pain *vs.* the cost of longer operative time as describe by some authors [1,2]. Results of randomised studies are conflicting [3,4]. Laparoscopic procedures in general promise to improve the health related outcome [5,6]. Whereas various laparoscopic approaches have shown their superiority regarding the classic factors (wound infection, postoperative pain, return to normal activity), randomized studies, focusing primarily on the patients perspective (such as quality of life) are less often conducted and the follow up time of most studies stops after half year. For appendectomy only a few studies focusing on quality of life in adults are available, providing a short follow up time of two weeks or six month [7,8]. There is no study comparing long term quality of life for children after OA and LA [9]. With these facts in mind a subgroup analysis for children was performed from already published data [10]. Target of this comparative study has been to clarify how younger patients who underwent appendectomy assess the long term course of events.

## Methods

### Selection of patients

Between 1999 and 2001 158 children (age 2 to16 years) underwent appendectomy (OA n = 83, LA n = 75) at the same hospital and were analysed retrospectively. Patients with diagnostic laparoscopy followed by an incidental appendectomy were excluded. Only patients with the clinical diagnosis of acute appendicitis were selected for this study. Preoperative body mass index (BMI) of all patients was calculated.

### Surgical procedures

Both modes of operations were performed by the same nine surgeons. Every surgeon performed more than 100 operations in every technique before he participated in our study. The mode of operation was chosen depending on the preferences of the operating surgeon. All surgeons performed both operations. OA was conducted under general anaesthesia applying a standard Mc Burney laparotomy at the right lower abdomen with buried stump.

LA with three port technique was carried out as described before [11]. The mesoappendix was divided by diathermy and the base of the appendix was resected with a laparoscopic stapler (Covidien or Ethicon, Germany). The appendix was extracted; in cases of a progressed inflammation an endobag was used. Finally the incisions were closed on the fascial level by an absorbable suture.

In all cases a single shot antibiotic prophylaxis with cefuroxime was administered preoperatively. Depending on the intraoperative findings a postoperative antibiotic therapy with cefuroxime (if considered necessary in combination with metronidazole) was commenced.

The intraoperative irrigation of the abdominal cavity with 0.9% sodium chloride solution depended on the stage of inflammation, as well as the utilization of a drain.

### Postoperative treatment and measurement

The most frequently used postoperative analgesics were morphine and acetaminophen. Other morphine-derivative analgesics were usually not prescribed and if applied converted to morphine equivalent dose for analysis. Postoperative temperatures were routinely taken every morning orally. In case of repeated measurement the highest temperature during 24 hours was included for analysis. The white blood cell count (WBC) (physiological range from 4.5 to 10.5/nl) were counted by SE 900 (Sysmex, Germany) and the C-reactive protein (physiological range < 0.5 mg/dl) was measured by Analytic analyser 912 (Hitachi, Japan). These data were not measured for every patient every day. The frequency of these measurements depended on the clinical course.

Complications were divided into general complications that were not related to the surgical procedure and specific postoperative complications related to the operation procedure.

Wound infections were defined following the CDC (Centres of Disease Control and Prevention) definitions of surgical site infections (SSI) (modification 1992) [12]. Haematoma and seroma categorization followed the ultrasound criteria if there were no signs of infection to be detected in the patient. Patients returning to the hospital with complications following appendectomy during the first four weeks were included in the analysis.

### Short term outcomes of the study

Investigated outcomes were operative time, required analgesics and postoperative complications within the first 30 days.

### Long term outcomes of the study

In 2008 a quality of life questionnaire (Short-Form 36 Health Survey, SF 36) was distributed to all operated patients by mail. This questionnaire was supplemented by additional questions regarding the appendectomy such as satisfaction with the size and appearance of the scar(s), as well as the quality and intensity of pain. Additionally, patients were asked whether they would recommend the operation method to family members or friends. The original version of this questionnaire was published in [10]. Answers were evaluated using a point score: not at all = 1, few = 2, moderate = 3 and very = 4. A control question was asked twice in order to ensure the reliability of the answers. Additionally, body weight and height were monitored in order to evaluate the BMI at follow-up.

We analysed primarily the summarizing question regarding the recommendation of the experienced method. Moreover, the remaining questions of the self-developed supplement regarding the operation method and the eight scaled scores of SF-36 (physical functioning, physical role functioning, bodily pain, general health perceptions, vitality, social role functioning, emotional role functioning, mental health) were evaluated.

### Statistical analysis

For short term outcomes the statistical analysis was performed using the Wilcoxon rank sum test, t-test (if normality could be assumed) and Pearson’s χ^2^ test for categorical data.

A subgroup analysis was performed for children between 2 and 10 years and adolescents from 11 to 16 years. This discrimination addressed the question, whether any differences occurred depending on the patients’ age. The evaluation was performed with Sigma Plot® (Version 11, Systat Software Inc.).

For long term outcomes patients differing more than one point in their answer to the control question (Q16 and Q23) were excluded from further analyses (two patients). An additive unweighted score was used for the two questions concerning the appearance and the size of the scar (Q13 and Q14, minimum 2, maximum 8), and answers were categorized into two groups (2–5 and 6–8).

The answers to the three questions concerning pain (Q20, Q21 and Q22) were summarized in an additive unweighted score (minimum 3, maximum 12), and answers were grouped into two categories (3–7 and 8–12).

Categorical outcomes were compared between laparoscopic and open appendectomy by Pearson’s χ^2^ test. For the comparison of the BMI values, age and the scores of SF-36, a Wilcoxon rank sum test was applied. The statistical calculations were performed using the statistical program SPSS 15.0 for Windows® (Version 15.0.1).

All performed tests were two sided and a *p*-value smaller or equal to 0.05 is considered statistically significant.

This retrospective study is of exploratory nature and therefore no adjustment for multiple testing is applied. Results have to be verified in an additional prospective randomized double blinded study.

### Ethics

This investigation was carried out in compliance with the Declaration of Helsinki. Laparoscopic and open appendectomies are part of standard surgical treatment without change in standard operating procedure and therefore did not require ethical approval. According to the Hamburg Hospital Law (Hamburgisches Krankenhausgesetz) the utilization of anonymized patient data for scientific research is part of the treatment agreement. This applies for a retrospective analysis of short term results as well. Written informed consent provided by the participants has been obtained prior re-evaluation. A counselling of the ethic committee (University of Kiel) was performed. For patients younger than 18 years the parents were consented as well and it was made clear for all participants that their participation is on voluntary basis.

## Results

### Short term results

Eighty-three children (34 female and 49 male), median age 11 years were treated by OA. Seventy-five patients (48 female and 27 male), median age of 12 years underwent LA. Both groups were not significantly different in sex-ratio and age (Table 1). Five patients (2 female and 3 male) in the laparoscopic group were converted to open appendectomy due to intraoperative findings (conversion rate 6.6%). Conversions were necessary due to technical difficulties during the procedure. All cases of conversion were assigned to the laparoscopic group.

**Table 1:** **Demographic data open *vs. *laparoscopic appendectomy**

**Parameter**	**Open appendectomy (n = 84)**	**Laparoscopic appendectomy (n = 75)**	***p *Value**
Female : male	34 : 49	48 : 27	0.124
Age [years]			
Median (range)	11 (3–16)	12 (5–16)	0.340
Operation time [min]			
Median (range)	38 (14–92)	30 (11–90)	0.006
BMI [kg/m^2^]			
Mean ± SD	17.4 ± 3.2	20.4 ± 3.3	<0.001

The degree of inflammation in both groups was equal in both groups without statistical significance (data not shown).

The median operative time (from skin incision to the end of closure) was significantly shorter for the laparoscopic (30 min) versus open procedure with 38 min (*p* = 0.006, Table 1). A subgroup analysis for perforated appendicitis showed a comparable length of operation in both groups (49 min open vs. 48 min laparoscopic procedure; *p* = 0.792).

Regarding required postoperative analgesics no differences for opiates and NSAID could be determined. The postoperative course of the available standard clinical inflammatory parameters (C-reactive protein, WBC and postoperative body temperature) was comparable for both procedures (data not shown). The rate of general postoperative complication was 2.4% in the open group (one urinary tract infection, one postoperative pancreatitis) and 2.6% in the laparoscopically operated group (one urinary tract infection and one thrombophlebitis of the arm). Both complications occurring in the laparoscopic group were conversions to OA. The rate of specific complications was 9.3% in the laparoscopic group and 10.7% in the open group (*p* = 0.778, Table 2).

**Table 2:** Specific minor and major postoperative complication (within the first 30 days)

**Minor complications**	**Open appendectomy (n = 84)**	**Laparoscopic appendectomy (n = 75)**	***p *Value**	**Thereof conversions**
Superficial Incisional SSI	1 (1.2%)			
Deep Incisional SSI	2 (2.4%)			
Haematoma	1 (1.2%)	2 (2.6%)		1
Port side hernia				
5 mm port		1 (1.3%)		
12 mm port		1 (1.3%)		
∑	4 (4.8%)	4 (5.3%)		1
Major complications				
Organ/Space SSI	1 (1.2%)			
Ileus	2 (2.4%)	1 (1.3%)		1
Relaparotomy	2 (2.4%)	1 (1.3%)		1
Bleeding		1 (1.3%)		
∑	5 (6%)	3 (3.9%)		2
Total	9 (10.7%)	7 (9.3%)	0.778	3

We also compared children (≤10 years) and adolescents (>10 years) concerning the benefit of either operative technique. In both groups we found a significant shorter operative time for the laparoscopic procedure without an increased complication rate. The other parameters did not show any significant differences (Table 3).

**Table 3:** Comparison of younger and older children

	**Open appendectomy**	**Laparoscopic appendectomy**	***p *Value**
Children 3–10 years			
Number of patients	59	29	
Median operative time [min]	38	32	0.018
Median morphine dose [mg]	0.5	0	0.0607
Median acetaminophen dose [g]	1	1	0.407
Children 11–16 years			
Number of patients	24	41	
Median operative time [min]	36	27	0.023
Median morphine dose [mg]	0	0	0.962
Median acetaminophen dose [g]	1.125	2	0.480

### Long term results

**Figure 1:**
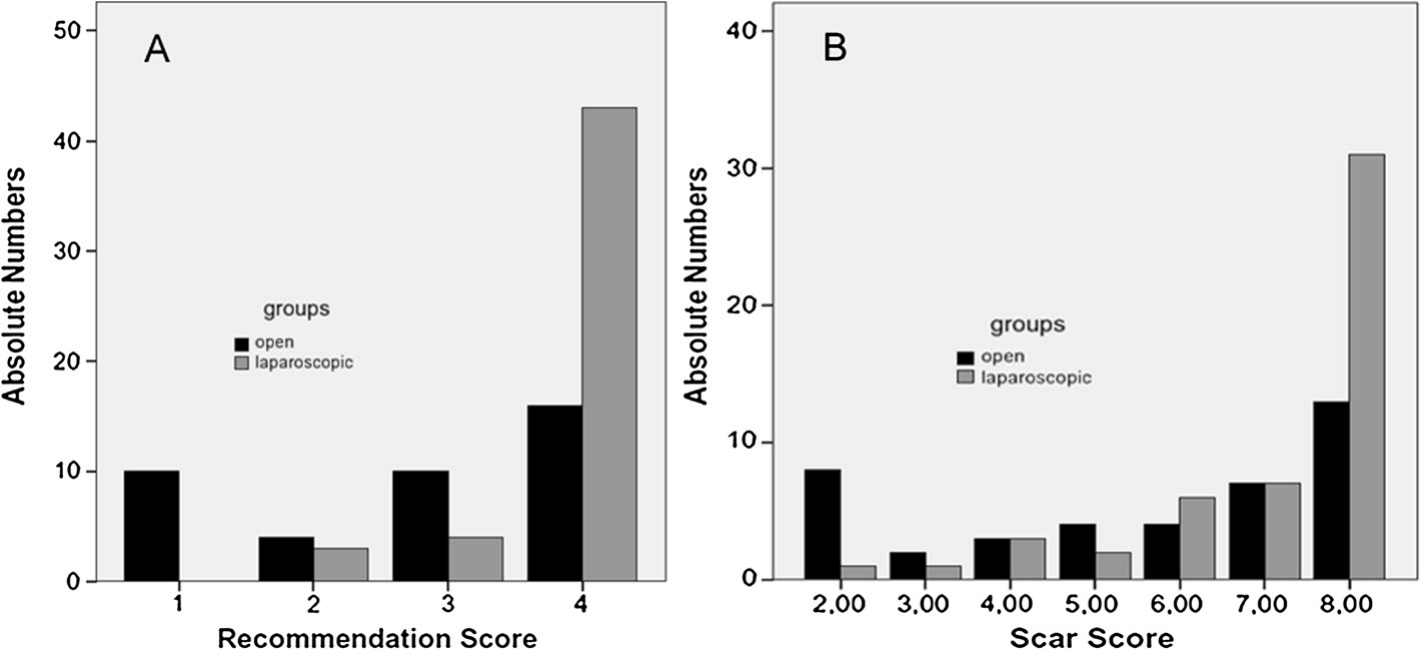
**Readiness and satisfaction. A)** Readiness to recommend the experienced operation procedure to relatives and friends; 1: not at all, 2: few, 3: moderate, 4: very. **B)** Postoperative satisfaction with appearance and size of the scar: 2: minimal satisfaction, 8: maximal satisfaction.

Only fully completed and returned questionnaires were included in this evaluation. The re-evaluation rate after a median of seven years (range 5.5 - 8.2 yrs) in both groups was 59% (Table 4). Evaluation of the primary outcome showed that significantly more patients of the laparoscopic group would recommend this operative procedure to family members or friends than those of the open group (Figure 1A; *p* < 0.001). For the secondary outcomes there was a significantly higher satisfaction of the patients of the laparoscopic group concerning size and appearance of scars (*p* = 0.004; Figure 1B).

**Table 4:** Results of the re-evaluation

**Parameter**	**Open appendectomy (n = 83)**	**Laparoscopic appendectomy (n = 75)**	***p *value**
Answered questionnaires [n]	42	51	
Female : Male [n]	19:23	34:17	0.868
Evaluation rate [%]	51	68	0.263
Age [years] at time of re-evaluation median (range)	19.1 (13.8-23.2)	18.5 (12.6-22.8)	0.195
BMI [kg/m^2^] median ( range)	22.9 (15.6-48.0)	22.9 (17.9-36.7)	0.431
Postoperative interval [years] median (range)	7.2 (5.7-8.2)	6.5 (5.5-7.2)	0.094

**Figure 2:**
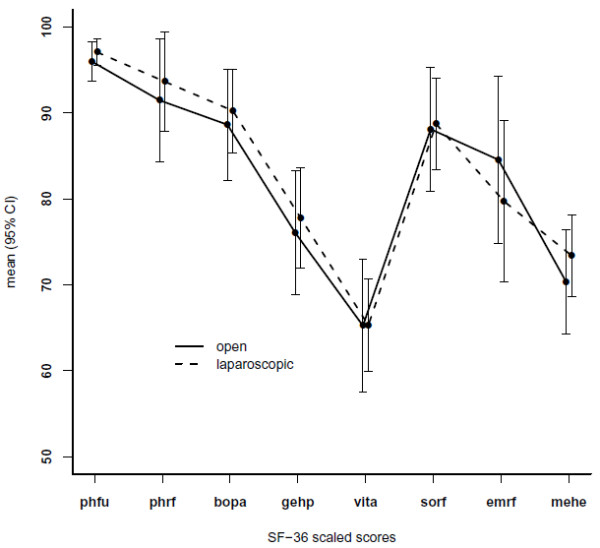
**Results of the SF-36 questionnaire in the OA and LA groups.** Phfu: physical functioning, phrf: physical role functioning, bopa: bodily pain, gehp: general health perceptions, vita: vitality, sorf: social role functioning, emrf: emotional role functioning, mehe: mental health, CI: confidence interval.

Results of the evaluation of SF-36 in the eight categories are shown in Figure 2. The results are very similar for the two groups and no significant differences were found (physical functioning p = 0.597, physical role functioning *p* = 0.340, bodily pain *p* = 0.899, general health perceptions *p* = 0.734, vitality *p* = 0.759, social role functioning *p* = 0.877, emotional role functioning *p* = 0.441, mental health *p* = 0.552).

Regarding the long term complications only one patient (OA) needed a reoperation due to a late intraabdominal abscess. A second OA patient suffered from an incisional hernia and required hernia repair (long term complication rate 5%). In the laparoscopic group two patients needed a re-laparoscopy due to intraabdominal adhesions. Additionally, one patient who was converted from LA to OA suffered from an incisional hernia and required a re-operation. Therefore a long term complication rate of 6% was calculated for the laparoscopic group.

## Discussion

Discussion about the superiority of LA versus OA is as old as the laparoscopic procedure itself. In the meantime the advantages of the laparoscopic procedure in adults seem to be accepted [13]. In children the relevance of LA is still on debate [3]. Previous studies showed several disadvantages of LA in children: longer operative time, increased risk for intraabdominal abscess etc. [1,14,15]. However, meta-analysis and large database analyses were unable to confirm these findings [16]. Beside this, large cohort studies showed a rapid increase of the proportion of laparoscopically performed appendectomies in children over the last 10 years: the frequency of LA increased up to 50% [15,17]. This correlates with the data presented here showing an increase of the laparoscopic proportion from 5% at the beginning of the evaluation to 75% at the end.

The proportion of perforated appendicitis in the study presented here is with 14 vs. 12% lower than in large cohort studies [15] but is comparable with other published studies [3]. Furthermore, the conversion rate of 6.6% in our study is similar to other retrospective and randomised studies in children [3]. The same holds true for early postoperative complications. Our rate of complications is being also comparable with large database analyses and multicentre analyses for children [15,18].

An often applied argument against LA is the longer operative time [19]. Compared to published studies focussing on children the operative time in our study has to be considered as quite short. The median operative time for LA is with 30 min significantly shorter than the operative time for OA (38 min). The clinical relevance of this difference however, is only of minor importance [20].

The early postoperative results of this study are not the primary endpoint of this study and were only described to show that this is a representative study population which is comparable to published studies regarding the primary complication rate [15,17].

Instead, our primary endpoint is the long term quality of life. The question investigated here is how operated children would apprehend possible constrictions following OA or LA and judge those in a different manner. It is interesting that, while for other laparoscopic procedures quality of life comparisons exist, for comparison of LA *vs.* OA in general only two studies could be retrieved evaluating this fact. Unfortunately, these studies included only patients older than 14 or 16 years [7,8]. Furthermore, these studies evaluated only the first half year after operation. This is a short period of time compared to our seven years re-evaluation period. We applied the SF-36 questionnaire an established tool for evaluation of quality of life [9,21,22]. This tool is applicable for children up to 14 years [23,24]. Even younger children are able to provide valid answers in such as questionnaires [25]. The differences between the laparoscopic and open operative procedure for appendectomy are not significant in this study as both operations are comparable with respect to all eight investigated scores of the SF-36 which is in accordance with other studies comparing laparoscopic and open procedure. In general measurable early postoperative advantages of the laparoscopic procedure appear to decrease over time [26,27].

Evaluation of questions concerning the operation directly shows a significantly higher satisfaction of the patients in the laparoscopic group concerning size and appearance of scars. The disposition to recommend the laparoscopic procedure to family members or friends may be based on this higher level of satisfaction with the scar.

The number of long term complications after seven years are equal in both operation groups since two patients in the open group and three patients in the laparoscopic group reported complications. Therefore, it may be considered interesting that intestinal adhesions were the main reason in the laparoscopic group. This matches with other findings which report that laparoscopic procedures do not reduce clinical relevant adhesions connected with pain or bowel obstruction [28].

Regarding the results of this study it should be taken into account that this is a retrospective study. No randomization was applied and the choice of operation method depended on the preferences of the surgeon. Nevertheless, randomized trials seldom report long term clinical outcomes such as quality of life. So, retrospective analyses can also give valuable information on postoperative quality of life [9]. In general retrospective analyses are included in meta-analyses in children given the limited availability of data [19].

The limited recovery rate of 59% may be seen as a further limitation of this study. Evaluating this recovery rate one has to keep in mind that even large data base analyses for appendectomy in children do not achieve higher follow-up rates [15,17]. Even though our sample size of approximately 80 patients per group may appear small it is still sufficient to show statistical significances for large to medium effect sizes. In this context it has to be pointed out that large sample size analyses have to be interpreted carefully since those are able to show statistical significances for small effect sizes with marginal differences which may be clinically unimportant [20,29]. Same holds true for the question if girls in the long term judge the cosmetic benefit higher than boys. Given the sample size a possible clinical significance should be seen with care. Unfortunately this holds true for the question if patients who were children or adolescents at the time of surgery would state their current quality of life differently as well. This is the reason for not showing a detail analysis regarding these two interesting facts as part of this manuscript.

It may be seen as a problem of the presented study that the patients are not blinded and a bias in the provided answers cannot be fully excluded. There is the possibility that the perception of LA as a more modern procedure may have influenced the patients’ recommendation to family members and friends.

The long term results of this study correlate with other studies in adults regarding the quality of life for open *vs.* laparoscopic procedures. During the years after surgery the early postoperative advantages of the laparoscopic procedure minimize in comparison to the open procedure [26,27,30-32]. Only the cosmetic advantages experienced by the patient seem to remain, which would be an argument for the application of mini laparoscopic instruments (2.5 mm ports) or the use of single port techniques.

## Conclusions

To our knowledge this is the first study which investigated quality of life in childhood more than seven years after operation. Neither OA nor LA seems to have relevant influence on the quality of life in younger patients in a long term evaluation. The postoperative results regarding cosmetic aspects appear to be an essential factor in rating an operative procedure in long term follow-up. Patients showed a higher satisfaction with their scars after laparoscopic surgery. The obtained data should be confirmed by a randomized blinded study.

## Competing interests

The authors declare that they have no competing interests.

## Authors’ contributions

MK: data evaluation, manuscript preparation. AP: Data interpretation and manuscript preparation. AC: Statistical analysis and manuscript draft. All authors read and approved the final manuscript.

## Pre-publication history

The pre-publication history for this paper can be accessed here:

http://www.biomedcentral.com/1471-2431/13/154/prepub
